# Nucleolin Promotes Cisplatin Resistance in Cervical Cancer by the YB1-MDR1 Pathway

**DOI:** 10.1155/2021/9992218

**Published:** 2021-04-21

**Authors:** Jing Ke, Chunming Gu, Heyan Zhang, Yang Liu, Wenhao Zhang, Huiling Rao, Shan Li, Fuyun Wu

**Affiliations:** School of Basic Medical Sciences, Hubei University of Medicine, Shiyan 442000, China

## Abstract

**Purpose:**

Cervical cancer is the fourth most common cancer in women worldwide and is the main cause of cancer-related deaths in women. Cisplatin (DDP) is one of the major chemotherapeutic drugs for cervical cancer patients. But, drug resistance limits the effectiveness of cancer therapy. Nucleolin (NCL) is a nucleocytoplasmic multifunctional protein involved in the development of cancer. It has been reported that NCL may be a potential target for modulation of drug resistance. However, the precise molecular mechanisms are poorly understood.

**Materials and Methods:**

Human cervical cancer Hela cells and their cisplatin-resistant cell line Hela/DDP were used in this study. The protein level of NCL in cervical cancer cells was measured by western blot analysis. Hela cells and Hela/DDP cells were transfected with NCL overexpression plasmid or NCL siRNA separately. MTT and EdU assay were performed to evaluate the cell viability and sensitivity to cisplatin. The drug efflux function of MDR1 protein was assessed by intracellular rhodamine-123 accumulation assay.The promoter activity of MDR1 was assessed by using a dual-luciferase reporter assay.

**Results:**

We found that the protein level of NCL was elevated in Hela/DDP cells. Overexpression of NCL increased cervical cancer cell proliferation and attenuated the sensitivity to cisplatin. Overexpression of NCL increased Multidrug resistance (MDR1) gene expression and drug efflux. Our results demonstrated that NCL was highly related with cisplatin resistance in cervical cancer. NCL played an important role in MDR1 gene transcription through regulation of the transcription factor YB1.

**Conclusion:**

Our findings revealed the novel role of NCL in cisplatin-resistant cervical cancer and NCL may be a potential therapeutic target for chemoresistance.

## 1. Introduction

Cervical cancer is the fourth most common cancer in women worldwide and the second most common cancer in women living in less-developed regions [1]. Cisplatin-based chemotherapy is an important treatment option for cervical cancer, but resistance to cisplatin often results in chemotherapy failure [2, 3]. As so far, the mechanism of cisplatin resistance in cervical cancer is not clear. NCL is a multifunctional phosphoprotein involved in ribosome assembly, rRNA maturation, mRNA stability, and so on [4, 5]. It is mainly located in the nucleolus, but also found in the nucleoplasm, cytoplasm, and cell membrane [6]. Many studies have shown that NCL plays an important role in tumor development [7, 8]. NCL is upregulated in a variety of tumor cells and promotes the proliferation, invasion, and migration of tumor cells through its action on different cellular pathways [9, 10]. In recent years, it was found that the expression of NCL was significantly increased in etoposide- and mitoxantrone-resistant breast cancer cells [11]. Recent reports have demonstrated that high NCL expression promotes drug resistance in acute lymphoblastic leukemia [12].These results indicate that NCL may be involved in drug resistance of tumor cells, but whether NCL is involved in cisplatin resistance in cervical cancer has not been reported. Here, we explored the relationship between NCL and cisplatin resistance in cervical cancer. We found a new mechanism of cisplatin resistance mediated by NCL via stimulating YB1-induced MDR1 transcription. Our results indicated that NCL may be a potential drug-resistant target.

## 2. Materials and Methods

### 2.1. Plasmids

The human NCL gene was amplified by PCR from the cDNA of Hela cells and cloned into the pcDNA4/TO vector. To construct MDR1-luciferase reporter plasmid, the nt −201 to nt +43 fragment of the MDR1 promoter was inserted into the pGL3-basic vector at the KpnI and HindIII sites. Y-Box mutated MDR1-luciferase reporter plasmid was generated by overlap extension PCR using the primer.

  Forward: 5′-GGTGAGGCTGATCAACTGGGCAGGAAC-3′
  Reverse: 5′-GTTCCTGCCC AGTTGATCAG CCTCACC-3′

### 2.2. Cell Culture and Transfection

The Hela cells were cultured in Dulbecco's modified Eagle's medium (DMEM, Invitrogen) supplemented with 10% fetal bovine serum (Hyclone), 50 U/mL penicillin, and 50 *μ*g/mL streptomycin (Invitrogen) at 37°C with 5% CO_2_, and cisplatin-resistant cervical cancer cells Hela/DDP were treated with DDP at 0.5 *µ*g/ml in the complete medium to maintain their resistant phenotypes. Hela cells were transfected with pcDNA4/TO-NCL plasmid using lipofectamine 3000 (Invitrogen) based on the manufacturer's instructions. After 48 h, cells were selected with zeocin (200 *μ*g/ml) for one week, and then, the cells were used to assay proliferation.

### 2.3. Western Blotting

The whole cell lysates were extracted using RIPA buffer supplemented with protease inhibitors, and the concentration of isolated protein was determined using the BCA protein assay. Samples were resolved by SDS-PAGE and transferred to PVDF membranes. Membranes were blocked in 5% nonfat dry milk in TBST for 1 h at room temperature and then incubated with the primary antibodies overnight at 4°C, followed by Horseradish-Peroxidase-linked secondary antibodies for 1 h at room temperature. Protein signals were detected by using the ECL western blotting substrate. The following antibodies were used in immunoblotting: rabbit monoclonal antibodies against NCL, YB1, MDR1, and GAPDH purchased from Proteintech Group.

### 2.4. siRNA Transfection

To knockdown NCL expression, the double-stranded small interfering RNA against human NCL (target sequence: 5′-UUUCUCAAACGAAGUAAGCUUdTdT-3′) and nonspecific siRNA (target sequence: 5′-UUCUCCGAACGUGUCACGU-3′) (purchased from GenePharma Company Ltd.) were used for transient transfection. Hela/DDP cells were plated into a 6-well plate. After 24 h, cells were transfected with 5 *μ*g NCL siRNA or control siRNA with Lipofectamine 3000 reagent according to the manufacturer's instructions. Gene silencing was assessed after 36 h by western blotting.

### 2.5. Cell Proliferation Assay

Cell proliferation was assessed by both MTT and EdU assays. For MTT assay, 3000 Hela cells transfected with NCL vector or Hela/DDP cells transfected with siNCL were seeded into a 96-well plate for overnight. After 36 h, cells were treated with different concentrations of cisplatin for 24 h. MTT was added to each well and incubated for 4 h at 37°C, and then, 100 *μ*l dimethyl sulfoxide (DMSO) was added to each well with plate shaking for 30 minutes. Absorbance was read at 540 nm using a 96-well plate reader. For EdU assay, Hela cells transfected with NCL vector or Hela/DDP cells transfected with siRNA were seeded in 24-well plates. After treated with DDP for 24 h, cell proliferation was detected using the EdU Cell Proliferation Assay Kit (KeyGEN, China). The EdU positive cells were determined by fluorescence microscopy and calculated from five different fields. Each experiment was repeated at least three times.

### 2.6. Quantitative Real-Time PCR Analysis

Total cellular RNA was extracted using Trizol (Invitrogen) according to the manufacturer's instructions. First-strand cDNA was generated with the HiScript 1 Strand cDNA Synthesis Kit (vazyme, China).Quantitative real-time PCR was conducted using a HiScript one-step qRT-PCR SYBR Green Kit (vazyme, China) and performed on the Bio-Rad CFX96 TM Real-time system. Gene expression in each sample was normalized to GAPDH expression. Primer sequences were as follows: NCL, 5′-GATCACCTAATGCCAGAAGCCAGC-3′; 5′-CAAAGCCGCCTCTGCCTCCACCAC-3′; MDR1, 5′-TGACTACCAGGCTCGCCAATGAT-3′; 5′-TGTGCCACCAAGTAGGCTCAAA-3′; GAPDH, 5′-GGAGTCAACGGATTTGGT-3′; 5′-GTGATGGGATTTCCATTGAT-3′.

### 2.7. Intracellular Rhodamine-123 Accumulation Assay

Hela cells transfected with NCL overexpression or empty vector were plated at 1 × 10^5^/well in 12-well plates and incubated for 24 h, cells were treated with or without DDP (2.5 *μ*g/mL) for 12 h, and then, Rh123 was added at a final concentration of 10 *μ*g/ml and incubated with cells at 37°C for 30 min. Cells were harvested and resuspended with cold PBS. The fluorescence intensity of the cells was measured at 488/575 nm using the FACS Calibur flow cytometer (Becton Dickinson, USA).

### 2.8. Luciferase Reporter Assay

The pGL3-MDR1-promoter-luc plasmid or Y-Box mutated MDR1-luc plasmid and NCL overexpression plasmid or empty vector were cotransfected into Hela cells. After 24 h, cells were treated with cisplatin for 24 h. Luciferase activity was measured using the Dual-Luciferase Reporter Assay System (Promega).

### 2.9. Statistical Analysis

Data analysis was performed using GraphPad Prism 5. All results are expressed as means ± S. E. M. Differences between the two groups were determined using Student's *t*-test. *P* < 0.05 was considered statistically significant.

## 3. Results

### 3.1. NCL Is Involved in Cisplatin-Resistant Cervical Cancer Cells

**Figure 1:**
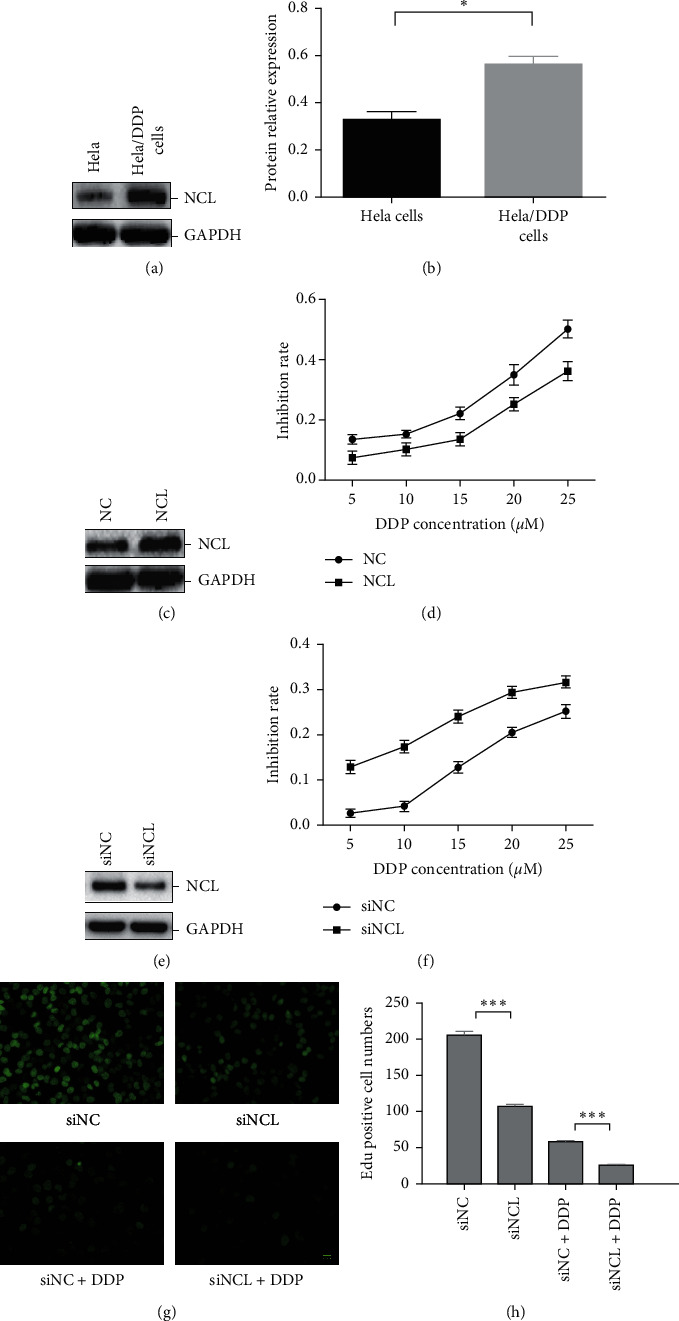
NCL is involved in cisplatin-resistant cervical cancer cells. (a) Western blot analysis and (b) quantification of NCL expression in cervical cancer, Hela cell, and cisplatin-resistant cervical cancer cell Hela/DDP. GAPDH was used as a loading control. ^*∗*^*P* < 0.05, compared with Hela cells. (c) The NCL expression level was detected by western blot in Hela cells transfected with NCL overexpression vector or empty vector. (d) NCL overexpression cells were treated with graded concentrations of DDP; then, cellular viability was assessed by MTT assay. (e) The same as that in (c), but Hela/DDP cells were transfected with NCL siRNA or control siRNA. (f) Same as that in (d), but with NCL knockdown cells. The data in the curves represent mean ± SD from three independent experiments. (g, h) Edu proliferation assay analysis of the effect of NCL knockdown on the growth of Hela/DDP cells treated with DDP. ^*∗∗∗*^*P* < 0.001, compared with control cells.

To determine whether NCL was associated with cisplatin resistance in cervical cancer, the expression of NCL was detected by western blotting in Hela cells and cisplatin-resistant cervical cancer cells Hela/DDP. As shown in Figures 1(a) and 1(b), NCL was expressed more abundant in Hela/DDP cells than in Hela cells. We then test the effect of overexpression and knockdown of NCL on the sensitivity to cisplatin of cervical cancer cells. NCL overexpression vector (NCL) or empty vector (NC) were transfected into Hela cells. Western blot results showed that the expression level of NCL was remarkably elevated in cells transfected with NCL overexpressing vector compared with the control (Figure 1(c)), and MTT assay result indicated that overexpression of NCL increased cervical cancer cell proliferation and attenuated the sensitivity to cisplatin (Figure 1(d)). Consistently, siRNA knockdown of NCL inhibited cell proliferation and enhanced the sensitivity of Hela/DDP cells to cisplatin (Figures 1(e) and 1(f)). We then performed the EdU assay, in which Hela/DDP cells transfected with siRNA were treated with or without DDP, then exposed to EdU (10 *μ*M) for 12 h, and visualized under a fluorescent microscope. As expected, knockdown of NCL attenuated the cell proliferation and enhanced the sensitivity of cisplatin (Figures 1(g) and 1(h)).These results suggested that NCL was involved in the cisplatin resistance to cervical cancer.

### 3.2. NCL Regulates MDR1 Expression

**Figure 2:**
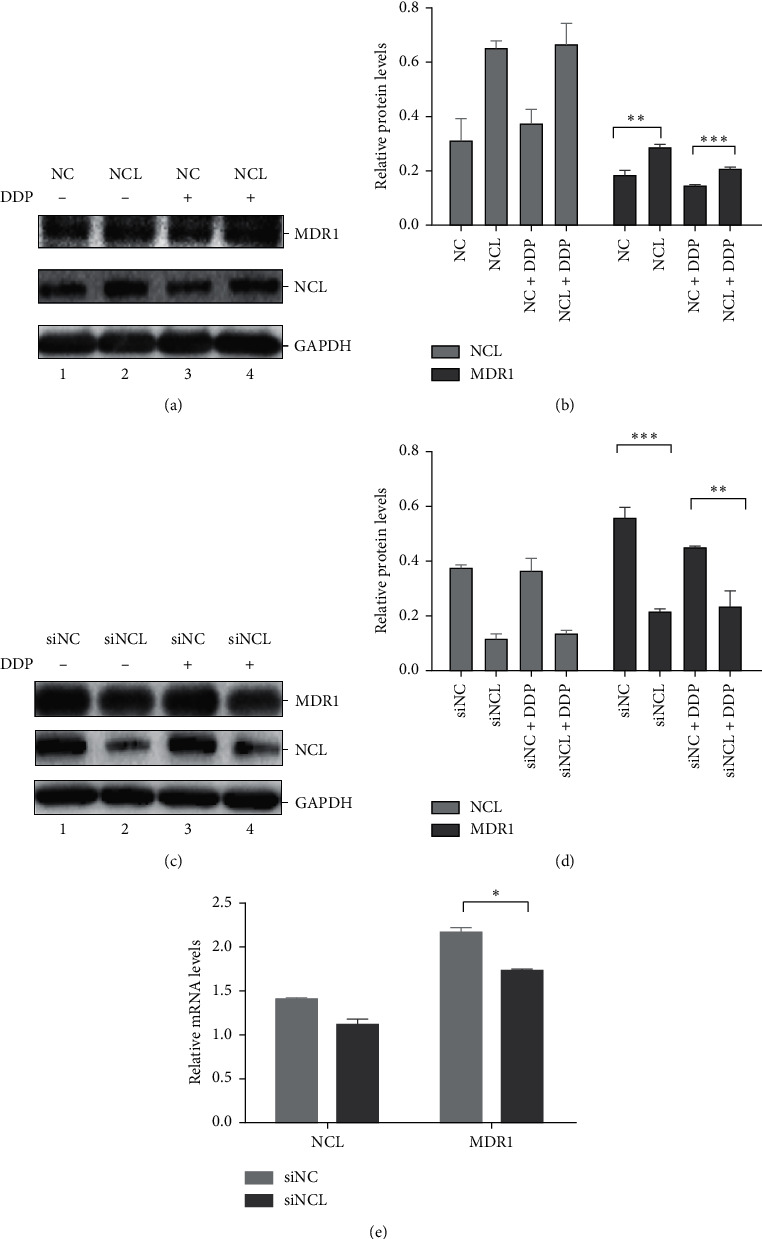
NCL regulates MDR1 expression. (a) Western blot analysis and (b) quantification of MDR1 expression in NCL overexpression Hela cells with or without DDP treatment. (c, d) The same as that in (a) and (b), but with NCL knockdown cells. (e) MDR1 mRNA expression in NCL knockdown cells was determined by qRT-PCR, and the mRNA levels were normalized to GAPDH.

Mechanisms of multidrug resistance in cancer are very complicated. One of the most important mechanisms responsible for multidrug resistance is the overexpression of multidrug resistance protein 1 (MDR1), also known as P-glycoprotein 1 (P-gp) or ATP-binding cassette subfamily B member 1 (ABCB1), which acts as an efflux pump for a wide spectrum of anticancer drugs. So, MDR1 proteins may be involved in the mechanism of cisplatin resistance mediated by NCL. To test this possibility, we detected the effect of overexpression and knockdown of NCL on MDR1 protein expression level. NCL overexpression plasmid or vector control was transfected in the Hela cells, and MDR1 and NCL were detected by western blotting. As shown in Figures 2(a) and 2(b), the MDR1 protein level was increased in NCL expressing cells, compared with that of cells expressing empty vector control. Conversely, a significantly decreased MDR1 level was observed after NCL knockdown cells when compared with the control (Figures 2(c) and 2(d)). Furthermore, we measured the mRNA level of MDR1 in the Hela cells transfected with NCL siRNA by qRT-PCR. Compared with the siNC group, the mRNA level of MDR1 was markedly decreased (Figure 2(e)).

### 3.3. NCL Overexpression Increases Drug Efflux

**Figure 3:**
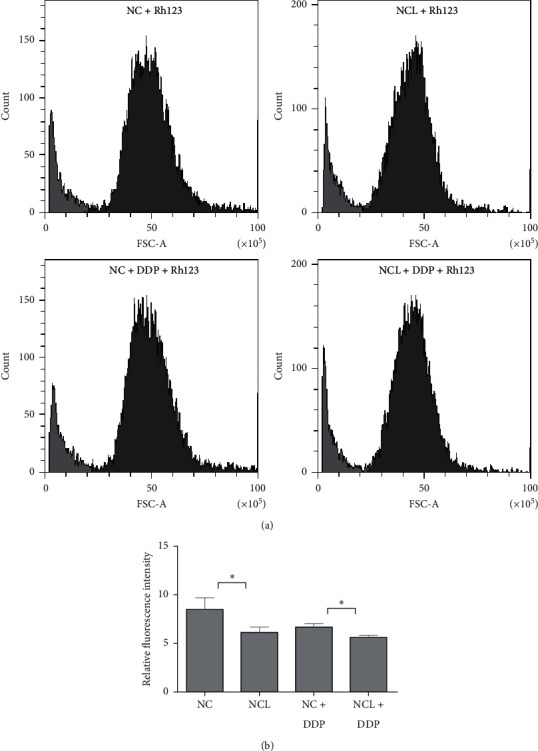
Rhodamine-123 (Rh123) efflux assay measuring MDR1 activity. (a) Flow cytometry analysis of intracellular accumulation of Rh123 in NCL overexpression cells with or without DDP treatment. (b) Mean fluorescence intensity of intracellular accumulation of Rh123. Results are mean ± SD of three independent experiments. ^*∗*^*P* < 0.05.

It has been reported that MDR1 functions as a drug efflux pump and extrudes multiple anticancer drugs contributing to multidrug resistance in many human cancers. So, we investigated whether overexpression of NCL affected the function of drug efflux. Rhodamine-123 accumulation assay was carried out by flow cytometry. As shown in Figure 3, the fluorescence intensity of rhodamine-123 was lower in NCL overexpression cells than control cells.

### 3.4. NCL Regulates Transcription Factor YB1 Expression

**Figure 4:**
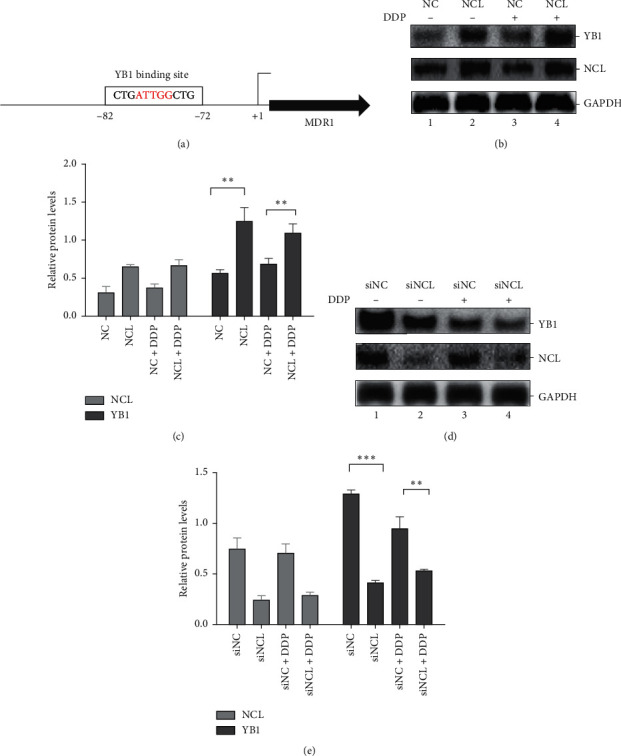
NCL regulates YB1 expression. (a) Schematic representation of human MDR1 gene promoter that contains a binding site for YB1. (b) Western blot analysis and (c) quantification of YB1 expression in NCL overexpression Hela cells with or without DDP treatment. (d, e) The same as that in (b) and (c), but in the NCL knockdown cells.

Transcriptional activation is generally accepted to be the principle mechanism for upregulating MDR1 gene expression. Many transcription factors play a role in the regulation of MDR1 gene expression. YB1 (Y-Box binding protein 1) has been demonstrated to be involved in multidrug resistance and regulation of downstream gene MDR1 (Figure 4(a)).So, we tested whether NCL may affect the expression of YB1. Results showed a significant increase of YB1 after NCL overexpression (Figures 4(b) and 4(c)).Similarly, NCL knockdown dramatically attenuated the expression of YB1 (Figures 4(d) and 4(e)).

### 3.5. NCL Regulates the Promoter Activity of MDR1 in an YB1‐Dependent Manner

**Figure 5:**
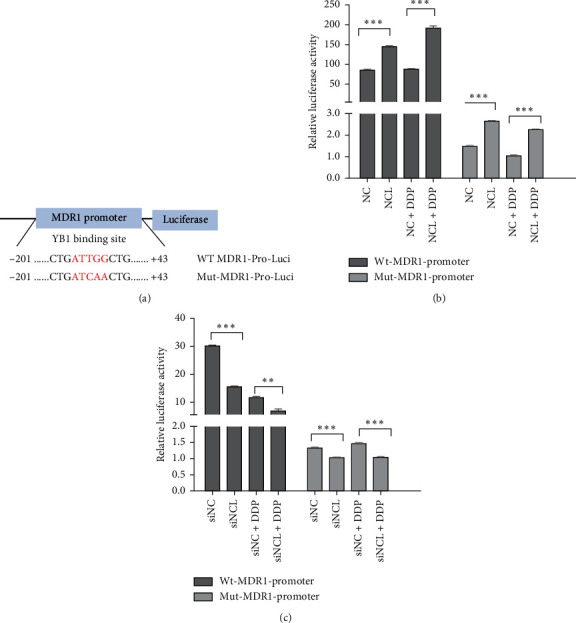
NCL regulates the promoter activity of MDR1 in a YB1-dependent manner. (a) Structures of the MDR1 promoter/luciferase reporter plasmid and Y-Box mutated MDR1-luc plasmid. (b) Functional analysis of MDR1 promoter activity by luciferase assays: NCL overexpression plasmid or empty vector were cotransfected with the wildtype or mutant MDR1 promoter reporter vector into Hela cells and then treated with or without DDP. After that, the cells were lysed and the luciferase activity was measured. Data are mean ± SD, *N* = 3. (c) The same as that in (b), but the Hela/DDP cells were cotransfected with siRNA and MDR1 promoter/luciferase reporter plasmid.

To further address the mechanisms that control MDR1 gene expression by NCL, firstly, we determined whether NCL modulated the activity of the MDR1 promoter. We constructed the MDR1 promoter/luciferase reporter plasmid and Y-Box mutated MDR1-luc plasmid (Figure 5(a)), then they were cotransfected with NCL overexpression vector or empty vector into Hela cells as described in Section 2. After treatment with or without DDP, luciferase activities were measured. As shown in Figure 5(b), MDR1 promoter activity was increased by about 2-fold after NCL overexpression compared with the control, and luciferase activity was markedly increased by about 4-fold following DDP treatment, while no change was observed in the NC group. As expected, NCL failed to activate luciferase activity in transfectants containing Y-Box mutated MDR1-luciferase vector. In contrast, MDR1 promoter activity was dramatically rEduced in NCL-silenced cells. Moreover luciferase activity was at a much lower level when cotransfected with Y-Box mutated MDR1-luciferase vector (Figure 5(c)). These results suggest that NCL regulates the expression of MDR1 by controlling its promoter activity in a YB1-dependent manner.

### 3.6. NCL Induces Cisplatin Resistance in Cervical Cancer Cells by the YB1-MDR1 Pathway

**Figure 6:**
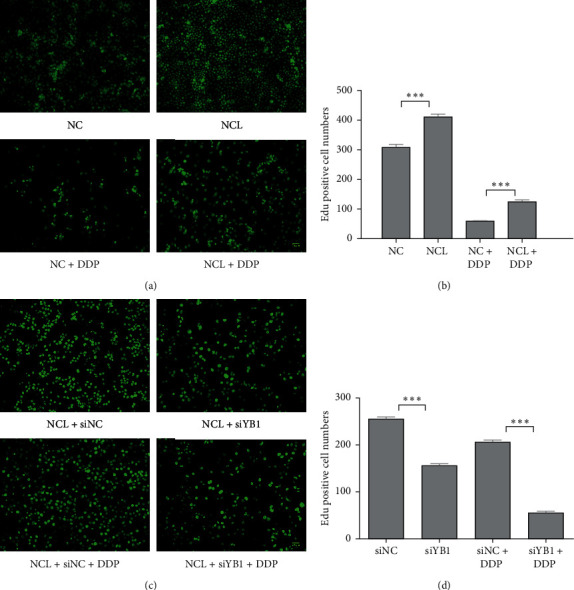
NCL induces cisplatin resistance in cervical cancer cells by the YB1-MDR1 pathway. (a) Edu proliferation assay analysis of the effect of NCL overexpression on the growth of Hela cells treated with DDP. (b) Quantification of Edu positive cell number. (c, d) The same as that in (a) and (b), but NCL overexpression cells transfected with YB1 siRNA or control siRNA. The results presented are representative of three independent experiments.

To further test whether NCL induces cisplatin resistance in cervical cancer cells by the YB1-MDR1 pathway, we also performed the EdU assay. As shown in Figure 6(a), NCL overexpression promoted cell proliferation and attenuated the sensitivity of cells to DDP compared with the NC control. However, a significant decrease in cell proliferation and increase in the sensitivity to DDP was observed after YB1 knockdown in NCL overexpression cells.

## 4. Discussion

Chemotherapy resistance is one of the main obstacles to successful clinical cancer therapy, and drug resistance can be obtained by different mechanisms, including drug efflux, apoptosis suppression, enhancing DNA repair, altering drug metabolism and persistence of cancer stem cells (CSCs), and epithelial-mesenchymal transition (EMT) [13–15]. Cisplatin is the major chemotherapeutic drug for cervical cancer, but cisplatin resistance has been a common and serious problem in the treatment of cervical cancer. Several reports have been published showing that overexpression of MDR1 gene enhancing drug efflux is associated with multidrug resistance in cervical cancer [16–18]. NCL, as a multifunctional protein with oncogenic properties involved in many key cellular processes, has attracted attention as a potential therapeutic target [19, 20]. NCL was able to traffic from the nucleus to the cytoplasm and cell surface, acting as a surface receptor for a variety of ligands implicated in tumorigenesis and angiogenesis. AS1411, an NCL-targeted DNA aptamer, has antiproliferative activity against a wide range of cancer cells [21–23]. It was reported that the NCL gene was upregulated in etoposide- and mitoxantrone-resistant breast cancer cells and associated with the drug resistance in acute lymphoblastic leukemia. However, the role of NCL in cisplatin resistance in cervical cancer is not clearly understood. In this study, we found that NCL was overexpressed in cisplatin-resistant cervical cancer cells and NCL expression was closely related to cisplatin sensitivity in cervical cancer cells. Overexpression of NCL significantly attenuated the sensitivity of cisplatin resistance in Hela cells, while NCL knockdown inhibited cell proliferation and reversed the cisplatin-resistant in Hela/DDP cell lines (Figure 1).Furthermore, we investigated the relationship between NCL and the expression level of MDR1 protein because one of the best characterized drug resistance mechanisms is overexpression of MDR1 which acts as a drug efflux and reduces the drug accumulation. We observed that knockdown of NCL expression with siRNA dramatically decreased MDR1 expression levels in protein and mRNA levels. Conversely, a significantly increased MDR1 level and intracellular accumulation of Rhodamine-123 were observed in NCL overexpression cells (Figures 2 and 3).It suggested that NCL contributed to the drug resistance by regulation the expression and function of MDR1. Transcriptional activation is the principle mechanism for upregulating MDR1 gene expression [24]. YB1, as a major transcription factor for the MDR1 gene, has been reported to regulate the expression of MDR1 and mediated multidrug resistance in a variety of tumors [25–27]. Here, we provided evidence that overexpression of NCL led to a markedly increased level of YB1 expression. Moreover, the results of luciferase reporter assay showed that NCL could modulate MDR1 promoter activity in a YB1-dependent manner (Figures 4 and 5). Furthermore, we demonstrated that NCL overexpression in cervical cancer cells led to cisplatin resistance depending on YB1 (Figure 6). Taken together, NCL is closely associated with drug resistance, so NCL inhibitor or the combination of NCL and MDR1 inhibitors may provide a potential therapeutic option for the treatment of multipdrug resistance in cervical cancer.

## 5. Conclusions

We found a new mechanism of cisplatin resistance mediated by NCL in cervical cancer. NCL contributed to the drug resistance by regulation the expression and function of MDR1 in a YB1-dependent manner. Our results indicated that NCL may be a potential drug-resistant target, and blocking its function may be a potential strategy to enhance the treatment efficacy in cisplatin-resistant cervical cancer.

## Data Availability

The data that support the findings of this study are available from the corresponding author upon reasonable request.
